# Recent advances in 2D hexagonal boron nitride (2D-hBN) applied as the basis of electrochemical sensing platforms

**DOI:** 10.1007/s00216-020-03068-8

**Published:** 2020-12-07

**Authors:** Alejandro García-Miranda Ferrari, Samuel J. Rowley-Neale, Craig E. Banks

**Affiliations:** Faculty of Science and Engineering, Manchester Metropolitan University, Chester Street, Manchester, M1 5GD UK

**Keywords:** 2D hexagonal boron nitride, 2D-hBN, Electroanalysis, Sensors, Electrochemistry

## Abstract

2D hexagonal boron nitride (2D-hBN) is a lesser utilised material than other 2D counterparts in electrochemistry due to initial reports of it being *non-conductive*. As we will demonstrate in this review, this common misconception is being challenged, and researchers are starting to utilise 2D-hBN in the field of electrochemistry, particularly as the basis of electroanalytical sensing platforms. In this critical review, we overview the use of 2D-hBN as an electroanalytical sensing platform summarising recent developments and trends and highlight future developments of this interesting, often overlooked, 2D material.

## Introduction

2D nanomaterials have attracted significant interest in a plethora of fields, particularly electrochemistry and electroanalysis, as can be observed by this article being part of this special issue. The first 2D nanomaterial that gained significant attraction and launched this field was graphene after Novoselov and Geim first isolated monolayer graphene and its interest has only grown since the award of their Nobel Prize in 2010, due to its reported extraordinary physical and chemical properties [1]. Nowadays, the term “2D nanomaterials” include more than a hundred different materials, including the family of carbon nanomaterials such as graphene, CNTs, fullerenes, graphene oxide, graphene QDs and other variants [2]. This has been rapidly extended to, for example, hexagonal boron nitride (2D-hBN, the so-called white graphene), transition metal chalcogenides (TMDs) (such as MoS_2_, MoSe_2_, WS_2_, WSe_2_) and homoatomic materials (such as antimonene, silicene, germanene, phosphorene) [3–11].

**Fig. 1:**
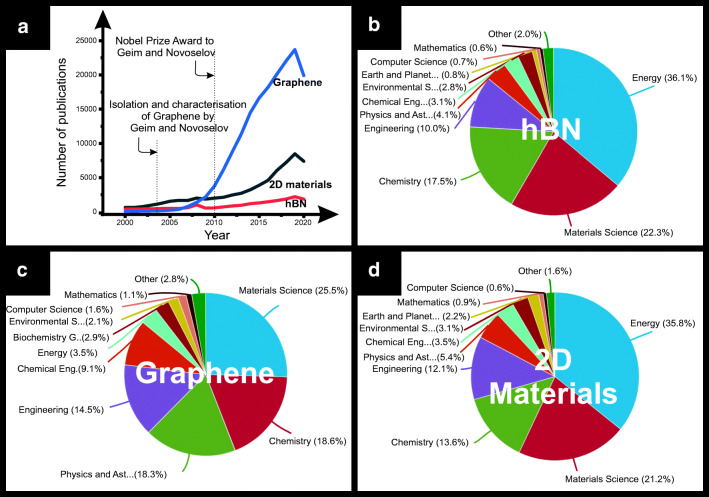
Number of publications and related fields to “graphene”, “2D materials” and “boron nitride” in the last 20 years. Data obtained from Scopus (accessed at the time of the submission of this paper)

A graphical representation of the exponential interest of research in graphene, 2D materials and boron nitride is depicted in Fig. 1, where the number of articles published between the years 2000 and 2020 is compared for the search terms “graphene”, “2D material” and “boron nitride”. It is clear upon inspection of Fig. 1 that the first isolation of pristine graphene in 2004, and then, the Nobel Prize Award in 2010 helped putting first graphene and then other 2D materials (including h-BN) in the spotlight of a wide variety of different research topics. To name a few, materials science, chemistry and physics are the main topics for graphene research, being energy, materials science and chemistry the main ones for both terms “2D materials” and “boron nitride”.

The application of electrochemistry in analytical methods investigates changes in electrical properties that are related to chemical reactions/parameters. Electroanalysis has been widely explored for quality control [12], water [13] and environmental monitoring [14, 15], forensics [16–18], food [19–21] and biomedical [22, 23] applications to name just a few. In the application of 2D nanomaterials, they have reported to have significant benefits over other nanomaterials in the field of electroanalysis [8, 24]. Monolayered 2D materials offer high surface-to-volume ratios, which enhances their chemical reactivity than the one exhibited by their bulk form (inert activity) [25, 26]. In this critical review, we overview the use of 2D-hBN as the basis of electroanalytical sensing platforms and summarising recent exciting developments and highlight potential future developments of this interesting material.

## 2D-hexagonal boron nitride in electroanalysis

**Fig. 2:**
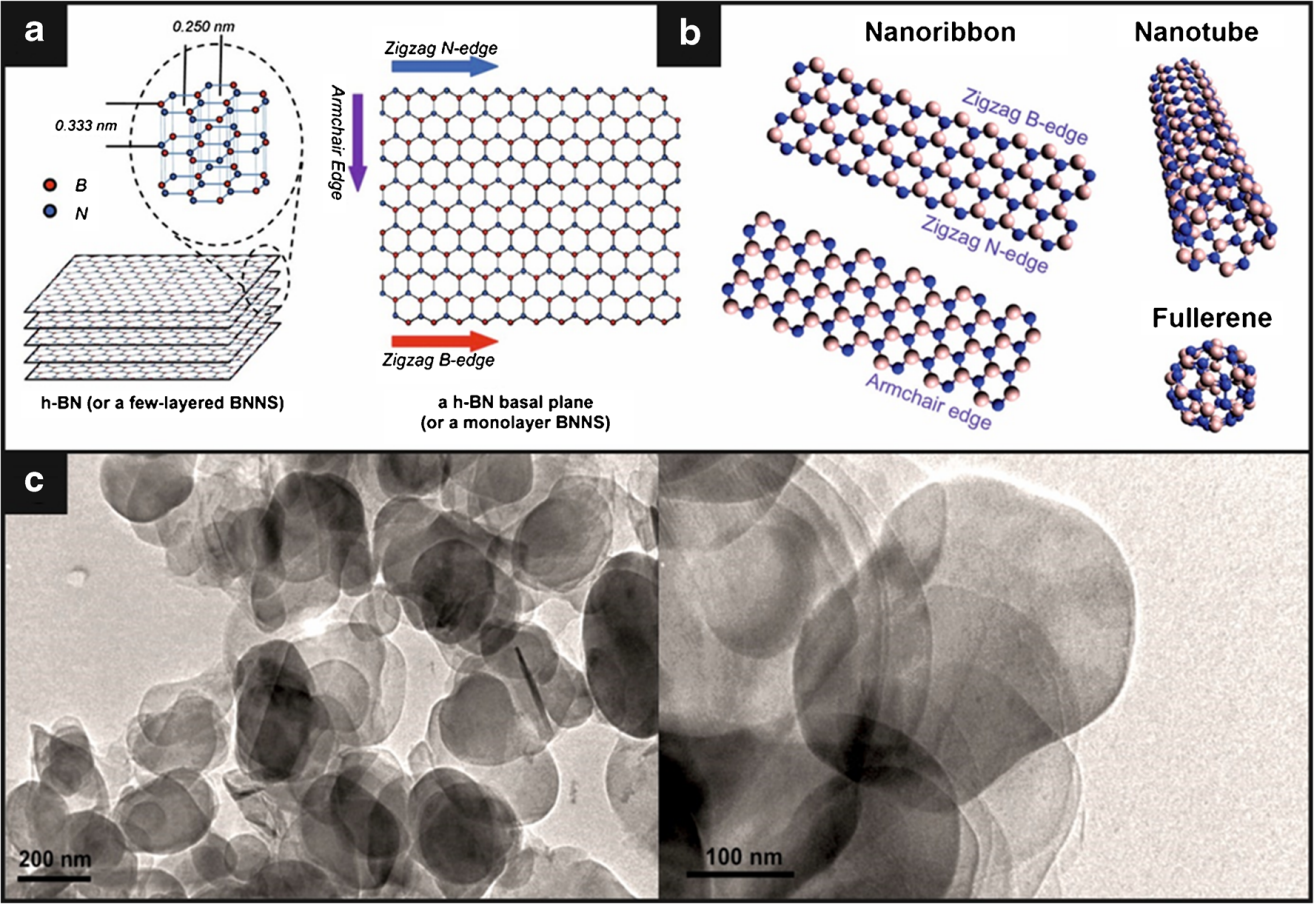
An overview of hexagonal boron nitride structures. Figures reproduced from **a** [27], **b** [29] and **c** [30]

2D-hexagonal boron nitride (2D-hBN) is a structural analogue of graphite which presents an sp^2^ hybridisation of B–N bonds in a layered honeycomb structure comprising rings of borazine (B_3_N_3_H_6_) [27]. Boron nitride (BN) is chemically stable exhibiting four well-known polymorphs: wurtzite, rhombohedral, cubic and hexagonal [28] (and references therein). Figure 2 overviews the various hexagonal boron nitride structures highlighting the intra- and inter-planar sizes and the edges of a nanosheet. Nanoribbons can be in either a zigzag (B- or N-edged) or armchair (BN pair edge) conformation, which are typically comprised of lateral sizes from a few hundred nanometres to tens of microns, depending on the various fabrication approaches employed. 2D-hBN layers can also stack on each other forming few- and multi-layers via van der Waals forces at a distance of 0.333 nm [27]. Other common structural forms are nanotubes, fullerenes and quantum dots.

**Fig. 3:**
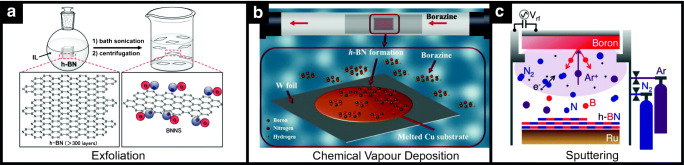
Schematic representation of boron nitride manufacturing methods: exfoliation (**a**), chemical vapour deposition (**b**) and sputtering (**c**). Reprinted from [37, 41, 42] with permission from the American Chemical Society and Elsevier respectively

Typically, 2D materials are fabricated via one of two routes: a bottom-up (BU) or a top-down (TD) method. TD approaches start from bulk materials as a starting point and transform it to achieve a monolayer. On the other hand, bottom-up (BU) approaches synthesise a pristine/monolayer from precursor(s). BU fabrication methods give larger yields of end products but do exhibit larger contamination (which might affect the electrochemistry of the material itself [31–33]), defects and lower quality of the nanosheets; on the contrary, TD lead to pristine materials but in lower quantities. In the case of hexagonal boron nitride, liquid-phase, ultrasonication-assisted, microwave-assisted, chemically assisted and mechano-chemical exfoliation methods are the most common BU approaches utilised within the literature [34–36]. In regard to BU methods, the most common are chemical vapour deposition (CVD) and physical vapour deposition (sputtering) [37–40]. Figure 3 includes a schematic representation of exfoliation (a), chemical vapour deposition (b) and sputtering (c) manufacturing methods for boron nitride from the literature [37, 41, 42]. The various fabrication approaches reported to 2D-hBN will not be covered in detail here, but we note that the fabrication route can affect the electrochemical performance of 2D-hBN (see, for example, the work by Khan et al. reporting the effect of surfactants in the electrochemical properties of 2D-hBN [43]).

According to the literature, 2D-hBN has a wide band gap of ca. 5.6 eV [44, 45]; therefore, it is usually reported as an electrical insulator [45, 46]. Theoretical simulations have been used to describe physical and chemical properties of multiple combinations of synthesis, characterisation and substrates with 2D-hBN [47–49]. From the electroanalysis perspective, researchers are in a continuous search of new electrode materials that offer a wide useful potential range, low background current, reproducibility and stability, and lastly fast electron transfer kinetics. Although 2D-hBN is commonly reported as non-conductive [49, 50], *it is* finding use as the basis of electroanalytical sensing platforms. Table 1 provides a summary of some recent advances of 2D-hBN applied to electroanalytical applications, and one can readily see that there is a limited amount of literature reporting the use of 2D-hBN as the main *active* material for this purpose.

**Table 1:** Overview of recent literature reports of 2D-hBN utilised as the basis of electroanalytical sensing platforms towards various (electro)analytical targets

Material	Analyte	Linear range	LOD	Reference
Au-NPs/2D-hBN/GCE	H_2_O_2_	0.04–50 mM	8.3 μM	[51]
Au-NPs/2D-hBN/GCE	Luteolin	10–400 pM	1.7 pM	[52]
MIP/Au NPs/2D h-BN/GCE	Diethylstilbestrol	5 pM–0.02 μM	0.1 pM	[35]
Au-NPs/2D-hBN/FTO	Myoglobin	0.1–100 μg mL^−1^	34.6 ng mL^−1^	[53]
MIP/Fe@Au NPs/2D hBN/GCE	Cypermethrin	10^−13^–10^−8^ M	0.03 pM	[54]
HMICI-Pt-NPs/POM/2d h-BN/CPE	N-Hydroxysuccinimide	0.1–300 μM	60 nM	[55]
MIP/GQDS/2D-h-BN/GCE	Serotonin	0.001–10 nM	0.1 pM	[56]
Trz-BN/GCE	l-Cysteine	-	-	[57]
Cu-h-BNNS/GCE	Nitrite	0.09–9853.45 μM	0.03 μM	[58]
2D-hBN-QDs	Vitamin C	0.8–5 mM	0.45 μM	[59]
D-2D-hBN/GCE	4-AP and Ph	0.01–30 μM (4-AP), 0.1–30 μM (Ph)	0.003 (4-AP) 0.035 (Ph) μM	[60]
2D h-BN/SPE	Dopamine	-	0.65 μM	[30]
2D h-BN whiskers/Ti	Nitrite	10–400 μM	0.089 μM	[61]
flake 2D-hBN/GCE	Vitamin C	30–1000 μM	3.77 μM	[62]
flake 2D-hBN/GCE	Dopamine	0.5–150 μM	0.02 μM	[62]
flake 2D-hBN/GCE	Uric acid	1–300 μM	0.15 μM	[62]
CN-hBN/GCE	Methyl parathion	0.0002–2 nM	0.001 nM	[63]
2D-hBN/f-MWCNTs/GCE	β-Agonists	0.001–10 nM	0.0001 nM	[64]
BN/graphene/GCE	Nicotine	1–1000 μM	0.42 μM	[65]
Bi_2_O_3_/h-BN/SPCE	Flutamide	0.48–87 μM	0.009 μM	[66]

*Au*, gold; *NPs*, nanoparticles; *GCE*, glassy carbon electrode; *MIP*, molecular imprinted polymer; *Fe*, iron; *FTO*; fluorine-doped tin oxide electrode; *HMICI*, 1-hexyl-3-methylimidazolium chloride; *POM*, polyoxometalate; *CPE*, carbon paste electrode; *GQDS*, graphene quantum dots; *Trz*, triazine; *Cu*, copper; *QDs*, quantum dots; *Ti*, titanium; *4-AP*, 4-aminophenol; *Ph*, phenol; *BNNS*, boron nitride nanosheets; *SPE*, screen-printed electrodes; *CN*, graphitic carbon nitride

Uosaki et al. [49] first reported the use of 2D-hBN upon gold (single crystal) electrodes as an electrocatalyst for the important electrochemical reaction, the oxygen reduction reaction (ORR) which was only possible on the gold electrode with no electrocatalytic effect observed upon glassy carbon electrodes. While the ORR was found to proceed to produce hydrogen peroxide, rather than the desired product of water, this work was seminal in demonstrating that there is an importance of 2D-hBN–substrate interaction and that 2D-hBN can be utilised in electrochemistry [49]. This work has been extended by others (see, for example, references [43, 67] and [68]).

Following the above seminal work, Khan et al. reported for the first time the utilisation of 2D-hBN as the basis of an electrochemical sensing platform using the example of the simultaneous electrochemical sensing of dopamine and uric acid determination via drop-casting the 2D-hBN onto screen-printed graphite macroelectrodes (SPEs) [30]. The electrochemical response was found to be highly dependent upon the interaction of the 2D-hBN and the underlying supporting electrode material and amount of deposited material, giving an electrocatalytic response in comparison to the (bare) underlying carbon electrodes. Using optimised condition, suitable peak resolution between dopamine and uric acid was found to be possible with competitive electroanalytical outputs. This manuscript was the first to report that the novel nanomaterial 2D-hBN is a beneficial electrocatalytic material for what might be considered an initially unlikely candidate. Such work has been extended by Li et al. [62] for the simultaneous sensing of vitamin C, dopamine and uric acid using flake 2D-hBN upon glassy carbon electrodes (GCE) which has a high density of defects and active surface groups resulting in wide linear ranges and low limits of detection and also exhibited anti-interference abilities.

Other reports of utilising 2D-hBN have been in electrode configurations with other nanomaterials in the form of nanocomposites. For example, Yola and Atar [56] utilised graphene quantum dots with 2D-hBN with molecularly imprinted polymers upon a GCE for the sensing of serotonin. This approach was shown to be (electro)analytically competitive over other literature reports with the authors attributing the enhanced outputs of their sensor due to charge transfer facilitated by the graphene quantum dots/2Dh-hBN, reduced mass transfer resistance on the underlying supporting electrode (GCE) and synergistic effect between the 2D-hBN and the graphene quantum dots [56]. Further inspection of their reported voltammetric responses [56], where a bare GCE is compared to a GCE electrode modified with 2D-hBN and graphene QDs/2D-hBN towards a simple redox probe, indicates a significantly differing voltammetry, which can be inferred as a change from diffusional (at the GCE) to thin-layer/adsorption type effects (at the 2D-hBN and graphene QDs/2D-hBN modified electrodes. The later gives rise to larger peak current/analytical response, which is expected [69]. This potentially contributed to the factors that the authors attributed to the improved electrochemical response, but further analysis would have been useful. The authors successfully utilised their nanocomposite sensor for the reliable measurement of serotonin in urine samples [56]. This avenue of using 2D-hBN in nanocomposites has been extended to the sensing of methyl parathion using graphitic carbon nitride, 2D-hBN and molecularly imprinted polymer [63]. This approach was shown to exhibit electroanalytical performances superior to previous literature reports and was shown to be successfully applied to orange juice samples for methyl parathion detection. In a similar vein, β-agonists have been shown to be electroanalytically detected in urine samples using 2D-hBN/multi-walled carbon nanotube nanocomposite modified GCE in the presence of ascorbic acid and uric acid [64]. Other approaches have combined 2D-hBN with graphene for the detection of nicotine applied in real tobacco samples [65] and 2D-hBN with bismuth oxide for the sensing of flutamine applied in environmental samples [66]. In all cases, the synergy of mixing the various components comprising the nanocomposite is attributed to the beneficial electrochemical response. Generally, authors *fail* to show data of the various ratios of the components comprising the nanocomposite upon the electrochemical/electroanalytical response demonstrating how they chose their final composition.

**Fig. 4:**
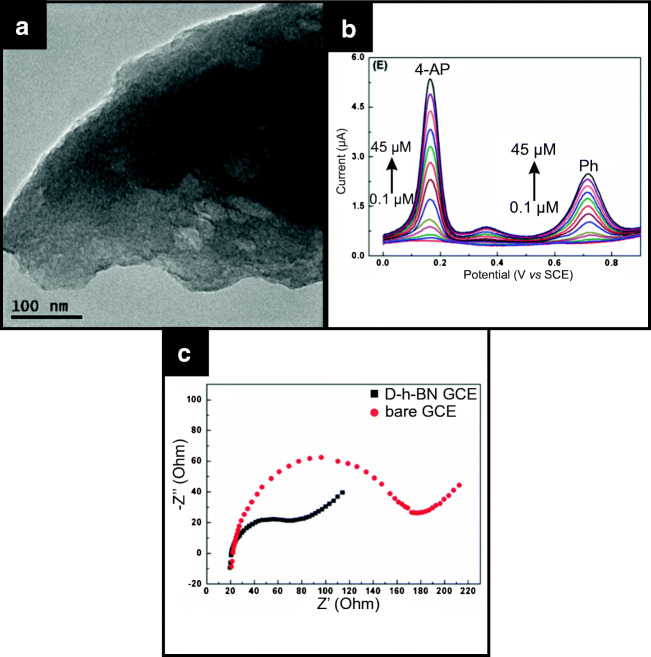
TEM image of defect-enhanced h-BN (d-h-BN) (**a**), simultaneous calibration plot (DPV) of 4-AP and Ph using d-h-BN/GCE (**b**) and electrochemical impedance spectra of d-h-BN/GCE (**c**). Reprinted from [60] with permission from Elsevier

Shen et al. have utilised defect-enhanced h-BN (termed (D-h-BN) on a GCE as a sensing platform for the detection of 4-aminophenol (4-AP) and phenol (Ph) [60] and lead [70]. They report the synthesis of defective h-BN via a single precursor calcination process. As shown in Fig. 4, defective h-BN exhibits the presence of pore holes within the basal plane of the typical laminar structure of 2D-hBN. These defects/holes are reported to be chemically active and provide electrochemical active sites via defect-related sub-levels in the band gap [60]. In both cases, the improved electrochemical response was compared to a bare GCE, which demonstrated the D-h-BN to give to superior electroanalytical signatures, attributed by the authors to be due to the material exhibiting fast electron transfer, large electrochemical active surface area and abundant electroactive sites, which were induced by the defective nature of the D-h-BN structure. The authors demonstrated the successful determination of 4-AP and Ph in tap and lake water samples.

**Fig. 5:**
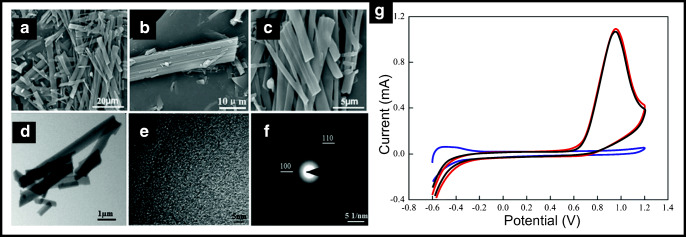
SEM and TEM micrographs of the h-BN whiskers. **a** Low-magnification SEM images; **b**, **c** high-magnification SEM images. **d** TEM images. **e** HRTEM. **f** The SAED pattern. Cyclic voltammetric curves (**g**) of the poorly crystallised BN whiskers electrode (red line), the highly crystallised BN whiskers electrode (black line) and bare Ti electrode (blue) towards the electrochemical oxidation of nitrite (1.0 m mol L^−1^ nitrite in 0.1 mol L^−1^ phosphate buffer). Reproduced from reference [61] with permission of the Royal Society of Chemistry

Luo et al. [61] reported the useful fabrication of hexagonal boron nitride (h-BN) whiskers. Their hBN whiskers were synthesised via a polymeric precursor methodology utilising boric acid (H_3_BO_3_) and melamine (C_3_H_6_N_6_) as raw materials with the precursors slowly heated in a tube furnace to high temperatures (1073–1273 K) in a flowing nitrogen/hydrogen (5% hydrogen) atmosphere. The fabricated h-BN whiskers are shown in Fig. 5, which are 0.5–3 μm in diameter and 200–500 mm in length. The authors sought to demonstrate the usefulness of their h-BN whiskers by exploring the electroanalytical sensing of nitrite. Figure 5 shows cyclic voltammetric curves of h-BN whiskers (poorly and highly crystallised) and compared to those of a bare Ti electrode. The choice of electrode gives the impression that the h-BN whiskers give rise to outstanding electrochemical signatures, even electrocatalytic, one might suggest. That said, nitrite can be readily electrochemically oxidised using a range of carbon electrodes [71–75]. It would have been insightful to compare the electrochemical response of the h-BN whiskers to other carbon-based electrodes under the same employed experimental parameters to demonstrate that they exhibit a large electrochemically area, as stated by the authors [61]. Additionally, it would have been insightful to explore different coverages to try and optimise the electroanalytical outputs; that said, the sensor was shown to allow the determination of nitrite at various concentrations within tap water [61].

**Fig. 6:**
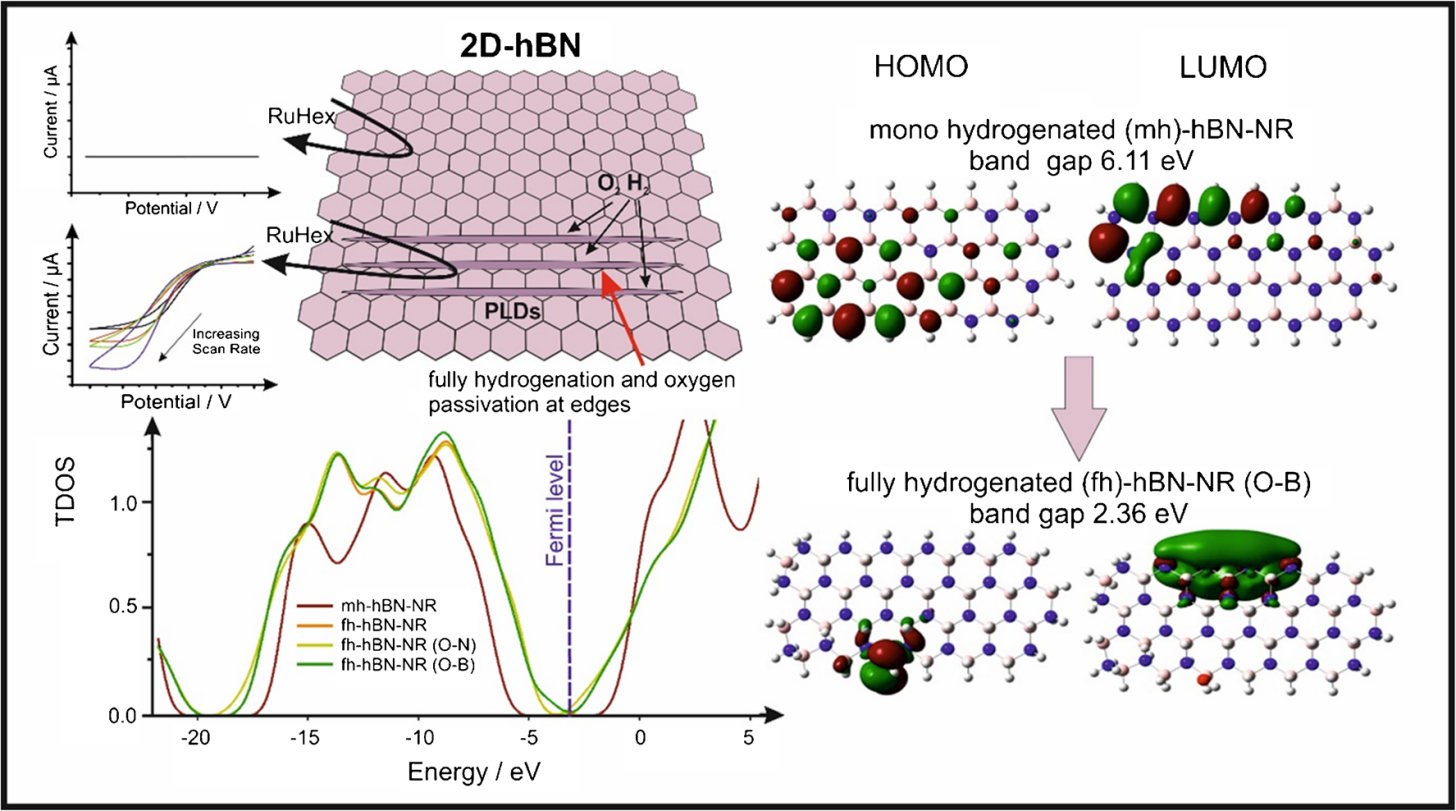
Graphical representation of physical linear defects (PLDs) upon 2D-hBN, giving rise to electrochemically useful signatures supported with DFT theory. Adapted with permission by the Royal Society of Chemistry from reference [91]

From the above analysis and inspection of Table 1, it is clear that 2D-hBN is being beneficially used in the field of electroanalysis. A critical question from the above literature is, however, why does this initially overlooked material, reported to be an insulator, clearly functions as an electrochemical/electroanalytical sensing platform? As the literature has progressed, many infer their beneficial responses to be due to a range of factors, such as high surface area, increased electron transfer, high accessible active sites and fast electron transfer due to defects [43, 67, 76–91]. Many different surface morphologies have been reported, but *only* the work reported by Garcia-Miranda et al. [91] has shown the origin of electrocatalytic properties at true mono-layer 2D-hBN (CVD grown on Si/SiO_2_). As shown in Fig. 6, they compared the electrochemical signatures of monolayer 2D-hBN with that of 2D-hBN with physical linear defects (PLDs). In the former case, a pristine (no defects) 2D-hBN monolayer is utilised which gives rise to zero/negligible electrochemical outputs. This has probably been encountered previously and might have led to abandoning the use of this material in electrochemistry which, on first sight, agrees with literature reports of 2D-hBN being non-conductive. In the latter case, PLDs are introduced which transforms this previously electrochemically inert material into giving useful electrochemically signatures; these induced defects upon the basal plane of the 2D h-BN surface are the only source of the newly electroactive material and the reason of the change from insulator to semi-conductor. DFT calculations were used to calculate the band gap of the introduced PLDs, which reported that the fully hydrogenation and oxygen passivation of the created edges are capable of reducing the band gap from 6.11 to 2.36 eV. This insight explains why 2D-hBN utilised in the above studies (see Table 1) give rise to electrochemical/electroanalytical outputs, since a range of 2D-hBN materials are not true monolayer and have abundant edges and defects across their basal plane for electron transfer to occur; this will be further enhanced for multi-layers of 2D-hBN.

## Conclusions

In this review, we have demonstrated that the previously overlooked 2D-hBN is beneficially being utilised as the basis of electroanalytical sensing platforms. Tailored defect-rich boron nitride microstructures have great potential as active materials for electroanalytical sensor fabrication, rather than their conventional use as electrode substrates (i.e. monolayer 2D-hBN). Although the electrocatalytic behaviour of 2D-hBN is still relatively unexplored territory, with careful execution of defect incorporation into the boron nitride nanosheets, researchers are unveiling its novel sensing applications towards a variety of analytes. Based upon current literature reports, we provide a summary of areas that could potentially be explored/reported in future work utilising 2D-hBN and related structures: (1) exploring the lateral sizes, La and Lc and defects (at edges and across basal surfaces) and understanding how these parameters affect the electrochemical/electroanalytical responses and can be be tailored and utilised to optimise electrochemical outputs; (2) exploring the role of the underlying/supporting electrode; (3) undertaking and reporting coverage studies, such that optimisation of the electrochemical/electroanalytical responses can be achieved and also noting that thin-layer responses can be observed that might be mistaken for “electrocatalysis”; (4) exploring the synergy of various materials mixed together to form nanocomposites—which are the origin/dominated the electrochemical response and understanding how the various ratios of each material comprising the nanocomposite can give rise to optimal electroanalytical outputs. As research grows in the area of 2D-hBN electrochemistry, more parameters will likely need to be explored/unravelled but it is clear that this is an emerging and exciting field utilising an interesting and previously overlooked 2D nanomaterial.
